# Supplementary material for: Impact of the grassland ecological compensation policy on pastoral production efficiency—evidence from pastoral China
Source: PLoS One. 2025 Oct 17;20(10):e0330059. doi: 10.1371/journal.pone.0330059 (PMC12533914; doi:10.1371/journal.pone.0330059)
Supplement: S2 File — (DOCX) [file pone.0330059.s002.docx]

#Import data#

library(readxl)

> all <- read_excel("DEA.xlsx",

+ sheet = "1")

> View(all)

#Descriptive statistical analysis of relevant variables#

library(modelsummary)

datasummary(GI+LI+ROE+HI+TIS~Mean+SD+Min+Max+Var,data=all)

datasummary(policy+X1+X2+X3+

X4+X5+X6+X7+X8+X9+X10+X11+X12+X13~Mean+SD+Min+Max+Var,data=all)

#Rdea-related code#

library(rDEA)

dmu=1:468

#Define input, output and environmental variables#

Y = bootstrap_dea[dmu, c('HI','TIS')]

X = bootstrap_dea[dmu, c( 'GI', 'LI', 'ROE')]

Z = bootstrap_dea[dmu,c( 'policy','X1','X2','X3','X4','X5','X6','X7','X8',

'X9','X10','X11','X12','X13')]

#Hypothesis testing of returns to scale#

rts_input=rts.test(X=X, Y=Y,Z=Z, W=NULL, model="input", H0="constant",

bw="cv", B=100, alpha=0.05)

rts_input$pvalue

rts_input$H0reject

rts_input$H0level

#Bias-corrected data envelopment analysis with environmental variables#

XL= dea.env.robust(

X=X[dmu,], Y=Y[dmu,], Z=Z[dmu,], model="input",

RTS="variable",L1=100, L2=2000, alpha=0.01)

#The relevant results are output. For specific meanings, please refer to the rDEA description#

XL$delta_hat

XL$delta_hat_hat

XL$bias

XL$delta_ci_low

XL$delta_ci_high

XL$sigma_ci

XD= dea.env.robust(

X=X[dmu,], Y=Y[dmu,], Z=Z[dmu,], model="input",

RTS="constant",L1=100, L2=2000, alpha=0.01)

XD$beta_hat

XD$sigma_hat

XD$beta_ci

XD$sigma_ci

XD$delta_hat

XD$delta_hat_hat

XD$bias

XD$delta_ci_low

XD$delta_ci_high

XL$sigma_ci

all$SE_delta_hat<-XD$delta_hat/XL$delta_hat

all$SE_delta_hat_hat<-XD$delta_hat_hat/XL$delta_hat_hat

all$SE_bias<-XD$bias/XL$bias

all$SE_delta_ci_low<-XD$delta_ci_low/XL$delta_ci_low

all$SE_delta_ci_high<-XD$delta_ci_high/XL$sigma_ci

all$te1<-XD$delta_hat

all$te2<-XD$delta_hat_hat

all$pte1<-XL$delta_hat

all$pte2<-XL$delta_hat_hat

all$se1<-all$SE_delta_hat

all$se2<-all$SE_delta_hat_hat

XX= dea.env.robust(

X=X[dmu,], Y=Y[dmu,], Z=Z[dmu,], model="input",

RTS="constant",L1=100, L2=2000, alpha=0.05)

XX$beta_hat

XX$sigma_hat

XX$beta_ci

XX$sigma_ci

XG= dea.env.robust(

X=X[dmu,], Y=Y[dmu,], Z=Z[dmu,], model="input",

RTS="constant",L1=100, L2=2000, alpha=0.1)

XG$beta_hat

XG$sigma_hat

XG$beta_ci

XG$sigma_ci

#Robust test Tobit model#

library(AER)

tobit_model<-tobit(te1~policy+age+education1+skill+

totalpeople+mper+xie+esti+rent+xinsur1+sdis+area1+area2,right=1,data=dame4)

summary(tobit_model)

#Mediating effect test#

library(mediation)

model1<-tobit(te2~policy,right=1,data=all)

model2<-lm(zhong~policy,all)

model3<-tobit(te2~policy+zhong,right=1,data=all)

summary(model1)

summary(model2)

summary(model3)

#bootstrap test#

set.seed(1234)

result<-mediate(model2,model3,treat="policy",mediator="zhong",sims=1000, robustSE = T)

summary(result)

plot(result)

#The mediating effect test in the bruceR package#

library(bruceR)

PROCESS(all,y="te2",x="policy",mods="zhong")

#Test of the Mediating Effect of Large-scale Herders#

model4<-tobit(te2~policy,right=1,data=large)

model5<-lm(zhong~policy,large)

model6<-tobit(te2~policy+zhong,right=1,data=large)

summary(model1)

summary(model2)

summary(model3)

set.seed(1234)

result<-mediate(model5,model6,treat="policy",mediator="zhong",sims=1000, robustSE = T)

summary(result)

plot(result)

PROCESS(large,y="te2",x="policy",mods="zhong")

#Test of the Mediating Effect of Small-scale Herders#

model7<-tobit(te2~policy,right=1,data=small)

model8<-lm(zhong~policy,small)

model9<-tobit(te2~policy+zhong,right=1,data=small)

summary(model1)

summary(model2)

summary(model3)

set.seed(1234)

result<-mediate(model8,model9,treat="policy",mediator="zhong",sims=1000, robustSE = T)

summary(result)

plot(result)

PROCESS(small,y="te2",x="policy",mods="zhong")
