# Supplementary material for: Impact of the grassland ecological compensation policy on pastoral production efficiency—evidence from pastoral China
Source: PLoS One. 2025 Oct 17;20(10):e0330059. doi: 10.1371/journal.pone.0330059 (PMC12533914; doi:10.1371/journal.pone.0330059)
Supplement: S3 File — (DOCX) [file pone.0330059.s003.docx]

**Nearest Neighbor Matching (K=4)**

psmatch2 treated x2 x3 x4 x5 x6 x7 x8 x9 x10 x11 x12 x13,outcome(y) n(5) common

pstest x2 x3 x4 x5 x6 x7 x8 x9 x10 x11 x12 x13 ,both graph

psgraph

//Before match//

twoway(kdensity _pscore if _treated==1, legend(label(1 "Treated Group")))(kdensity _pscore if _treated==0, legend(label(2 "Control Group"))), xtitle("pscore") title("before match") scheme(slmono) name(g13)

//After match//

twoway(kdensity _pscore if _treat==1,legend(label(1 "Treated Group")))(kdensity _pscore if (_weight!=1&_weight!=.), legend(label(2 "Control Group"))),xtitle("pscore") title("After match") scheme(slmono) name(g23)

**Radius Matching (0.01)**

psmatch2 treated x2 x3 x4 x5 x6 x7 x8 x9 x10 x11 x12 x13,outcome (y) radius caliper(0.01) ate ties common odds

**Kernel Matching**

psmatch2 treated x2 x3 x4 x5 x6 x7 x8 x9 x10 x11 x12 x13,outcome (y) kernel kerneltype() ate ties common odds
