# Supplementary material for: Impact of the grassland ecological compensation policy on pastoral production efficiency—evidence from pastoral China
Source: PLoS One. 2025 Oct 17;20(10):e0330059. doi: 10.1371/journal.pone.0330059 (PMC12533914; doi:10.1371/journal.pone.0330059)
Supplement: Attachment — Submitted filename: Response to Reviewers.docx [file pone.0330059.s005.docx]

Response to Reviewer 1 Comments

**Title：Impact of the grassland ecological compensation policy on pastoral production efficiency—evidence from pastoral China**

**Manuscript ID:** PONE-D-25-40838

Thank you for your meticulous revisions of our articles in your busy schedule, which have helped us immensely in improving the quality of our articles. We have revised the manuscript in accordance with the comments and marked all the amends on our revised manuscript. The reviewer comments are laid out below in *italicized font* and specific concerns have been numbered. Our responses are listed in red font, the specific modified contents are listed in blue font, and the modified contents are highlighted in yellow in the manuscript.

***[Comments 1]：The paper summarizes existing research on the ecological and economic (income) effects of GECP, but it does not sufficiently review domestic and international studies on the impact of similar ecological compensation policies on production efficiency. The paper notes that research on GECP's effect on production efficiency is scarce and the conclusions are controversial, but it fails to conduct an in-depth exploration of the reasons for these controversies. The paper states, "while some studies have also found that ecological compensation is difficult to compensate for the increase in the cost of shepherding as a result of the policy, and the longer the time of the ban on animal husbandry, the greater the decrease in the efficiency of technological progress of the herding households," but it does not clearly elaborate on how the present study innovates upon or improves existing literature.***

**[Response 1]：**Thank you very much for your high-quality suggestions, they help us tremendously to improve the quality of our articles.In the literature review section, we supplemented the discussion on the impact of Payment for Ecosystem Services (PES) on production efficiency, deeply analyzed the root causes of the debate, and finally clearly expounded how this study innovates and improves on the basis of existing literature. The specific revised contents can be found in lines 91-106, 133-162 of the revised draft. The specific modifications are as follows:

A review of the literature on the implementation effects of payment for ecosystem services (PES) programs reveals two primary findings. First, the ecological impact of PES programs is notable. For instance, Thang (2021) employed microsurvey data to evaluate the environmental effects of Vietnam's PES program and reported that its implementation significantly improved natural forest conditions in the study area [10]. Conversely, Humberto (2023) utilized macro data to assess the effectiveness of PES programs in protecting forest cover in tropical regions of Mexico, revealing that the current PES methods were not highly effective [11]. Second, the economic impact of PES programs on the production activities of farmers and herders is significant. Seema (2023) demonstrated through a study of the economic benefits of a forest protection payment project in Uganda that PES programs play a crucial role in increasing the economic well-being of local residents [12]. Additionally, Orlando (2022) examined the relationship between PES programs and coffee production in Colombia and reported that PES programs have significantly improved the production efficiency of coffee producers [13]. Similarly, discussions on the implementation effects of the GECP in existing studies focus mainly on two aspects.

Reference:

[10]Thang Quyet Nguyen,Nguyen Tan Huynh,Wen-Kai K. Hsu. Estimate the Impact of Payments for Environmental Services on Local Livelihoods and Environment: An Application of Propensity Scores[J]. SAGE Open,2021,Vol.11(3).https://doi/abs/10.1177/21582440211040774

[11]Humberto Reyes-Hernández1. Payment for environmental services: Forest conservation and poverty alleviation in a tropical region of Mexico[J]. Land Use Policy,2023,Vol.133: 106847.https://doi.org/10.1016/j.landusepol.2023.106847

[12]Seema Jayachandran. The inherent trade-off between the environmental and anti-poverty goals of payments for ecosystem services[J]. Environmental Research Letters,2023,Vol.18(2): 025003.https://doi:10.1088/1748-9326/acb1a7

[13]Orlando RodríguezCA1,Maria Vrachioli2,Johannes Sauer3. Payments for environmental services and coffee production in Colombia: Technical efficiency across the world heritage status borders[J]. Ecological Economics,2022,Vol.200: 107531.https://doi.org/10.1016/j.ecolecon.2022.107531

In summary, although existing research provides important theoretical support for this paper, several deficiencies still exist: (1) The existing research focuses mainly on the impact of the GECP on the ecological environment and the lives of herders, and few studies have focused on the impact of the GECP on the pastoral production efficiency of herders. (2) There is still considerable controversy over the current conclusions concerning the impact of the GECP on the pastoral production efficiency of herders. (3) Few studies have analyzed the mechanism by which the GECP affects the pastoral production efficiency of herders from the perspective of grassland-scale heterogeneity. In view of this, the marginal contributions of this paper are reflected in the following three points: (1) By combining externality theory with technology induction theory, a theoretical analysis framework for the impact of the GECP on the pastoral production efficiency of herders is constructed, and the role of moderate-scale management in the relationship between the two is explored. (2) Based on field investigation data of herders in the pastoral areas of Inner Mongolia, China, the Two-Stage Double Bootstrap DEA model was used to calculate the pastoral production efficiency of herders, and the impact effect and impact path of the GECP on the pastoral production efficiency of herders were empirically tested. (3) The heterogeneity of the impact of the GECP on the breeding efficiency of herders with different grassland scales was explored. The aims of this study were to provide new ideas for increasing the pastoral production efficiency of herders, reducing the pressure on grassland ecological protection, improving the stability of herders' incomes and achieving high-quality development of animal husbandry.

***[Comments 2]：Although the paper mentions its main research content at the end of the introduction or literature review, its elaboration on the paper's inattentiveness or marginal contribution is not sufficiently clear or specific. It is recommended to supplement this further.***

**[Response 2]：**Thank you for your rigorous and constructive suggestions. We have provided a more detailed and specific elaboration on the innovation or marginal contribution of the paper. The specific revised contents can be found in lines 149-162 of the revised draft. The specific modifications are as follows：

In view of this, the marginal contributions of this paper are reflected in the following three points: (1) By combining externality theory with technology induction theory, a theoretical analysis framework for the impact of the GECP on the pastoral production efficiency of herders is constructed, and the role of moderate-scale management in the relationship between the two is explored. (2) Based on field investigation data of herders in the pastoral areas of Inner Mongolia, China, the Two-Stage Double Bootstrap DEA model was used to calculate the pastoral production efficiency of herders, and the impact effect and impact path of the GECP on the pastoral production efficiency of herders were empirically tested. (3) The heterogeneity of the impact of the GECP on the breeding efficiency of herders with different grassland scales was explored. The aims of this study were to provide new ideas for increasing the pastoral production efficiency of herders, reducing the pressure on grassland ecological protection, improving the stability of herders' incomes and achieving high-quality development of animal husbandry.

***[Comments 3]：The paper primarily uses cross-sectional data from a questionnaire survey, which has many limitations for this type of research. Please further elaborate and discuss these limitations.***

**[Response 3]：**Thank you for your valuable suggestions. In accordance with your opinions, we have further elaborated in detail on the limitations of cross-sectional data in the discussion section of the paper. Specific changes can be made to lines 789-803 in the revised draft.The specific modifications are as follows：

However, such data present certain limitations. First, the sparse population distribution in pastoral areas makes it challenging to obtain comprehensive survey data. This study includes only 468 sample herders from four leagues and cities in Inner Mongolia, resulting in a relatively limited sample size. Consequently, the empirical results may not fully represent the actual conditions of the entire pastoral area, and their reliability requires further verification. Second, cross-sectional data fail to capture the dynamic characteristics of variables over time and cannot reflect the longitudinal evolution of herders' behaviors and production efficiency. Therefore, the results of this paper cannot be used to make conclusions about the dynamic correlation and causal mechanisms between the GECP and pastoral production efficiency. Future research investigating the relationship between this policy and pastoral production efficiency should consider including additional representative pastoral areas in China, such as Qinghai, Tibet, and Xinjiang. By broadening the data coverage and increasing data quality, the representativeness and explanatory power of the research can be significantly improved.

***[Comments 4]：The descriptive statistics for the variables are rather simplistic. Typically, they should include indicators such as the sample size (N), mean, standard deviation, maximum, and minimum values.***

**[Response 4]：**Thank you for your careful review of this paper. Based on your feedback, we have provided a more detailed description of the variable. The specific modifications are as follows:

**Table 2. Description and Descriptive Statistical Table of the Influencing Factors and Variables of Breeding Efficiency.**

| **Form** | **Specific indicator** | **Indicator description** | **N** | **Mean** | **Standard deviation** | **Max** | **Mix** |
| --- | --- | --- | --- | --- | --- | --- | --- |
| Explained variable | Pastoral production efficiency | Value of integrated technical efficiency of pastoral farming in 2023 | 468 | 0.43 | 0.20 | 0.09 | 1 |
| Core explanatory variables | The total amount of compensation | Grass-animal balance incentive+ in 2023  Pasture rest subsidy in 2023 (ten thousand yuan) | 468 | 1.29 | 1.42 | 0.011 | 9.16 |
| Characteristics of the head of the household | The age of the head of the household | Age of household head (years) | 468 | 49.99 | 8.09 | 20 | 85 |
|  | the years of education of the head of the household | Years of schooling of household head (years) | 468 | 8.06 | 3.30 | 0 | 17 |
|  | Whether the head of the household has been trained in ranching technology | 1=yes, 0=no | 468 | 0.79 | 0.40 | 0 | 1 |
| household resource endowment | The size of the household | Total household size | 468 | 3.54 | 1.12 | 1 | 9 |
|  | The proportion of income from ranching | Farming income in 2023/  Total income in 2023 (%) | 468 | 0.78 | 0.23 | 0.03 | 1 |
|  | The number of herding machines | Number of farming machinery (units) | 468 | 3.61 | 2.59 | 0 | 16 |
| social resource endowment | Evaluation of socialized services in animal husbandry | 1=None, 2=Lower, 3=Average, the  4=better, 5=very good | 468 | 3.31 | 1.08 | 1 | 5 |
|  | Whether pastureland is rented | 1=Yes, 0=No | 468 | 0.81 | 0.29 | 0 | 1 |
|  | Whether the head of the household has animal husbandry insurance | 1=Yes, 0=No | 468 | 0.59 | 0.49 | 0 | 1 |
| location endowment | Distance to livestock exchange | Distance to nearest livestock trading market (kilometers) | 468 | 61.08 | 50.82 | 0 | 300 |
|  | Area dummy variable 1 | 1=Eastern, 2=Other | 468 | 0.26 | 0.44 | 0 | 1 |
|  | Area dummy variable 2 | 1=Central, 2=Other | 468 | 0.39 | 0.47 | 0 | 1 |

***[Comments 5]：The paper fails to discuss the potential endogeneity problem. The empirical results show a positive correlation between the ecological compensation policy and efficiency, but this may not be entirely a policy effect. It could partially stem from a selection bias, where "superior" herders (those with higher innate ability or better management skills) are both more efficient and receive more subsidies.***

[Response 5]：Thank you for your valuable suggestions. In response to your feedback, we have incorporated a discussion on endogenous issues within the robustness test section of the article. To mitigate potential sample self-selection bias, we employed the propensity score matching (PSM) method to further validate the benchmark regression results. The specific steps are as follows: First, we categorized the sample herders into a treatment group (those participating in the project) and a control group (those not participating in the project) based on whether the area of household contracted grassland exceeds 100 hectares. Subsequently, we conducted a propensity score matching analysis using Stata 17.0. Finally, we performed an empirical test based on the matched samples to assess the robustness of the estimated results after controlling for selection bias.Specific changes can be made to lines 565-620 in the revised draft. The specific modifications are as follows:

Endogeneity treatment

To correct the differences among samples and address the endogeneity issues caused by sample selection bias, in this study, the Propensity Score Matching (PSM) model is used to conduct an in-depth calculation and discuss the impact of the GECP on the pastoral production efficiency of herders.

When this method is employed, it is essential to divide the sample into treatment and control groups. However, the implementation of the Grassland Ecosystem Conservation Project (GECP) occurred simultaneously for all herders in China's major pastoral areas, thus precluding the existence of a natural control group. Consequently, in this study, existing research is used to select samples that are minimally impacted by the policy, thereby constructing a control group [49]. Specifically, herders with less than 100 hectares of contracted pastureland are included in the control group. This selection is based on the understanding that, within the study area, 100 hectares is typically considered the minimum pasture area necessary for a herder to sustain a basic livelihood. Field studies indicate that herders with pasture areas below this threshold tend to make relatively stable livestock production decisions due to livelihood pressures and are less affected, or even negligibly influenced, by policies, rendering them theoretically suitable for inclusion as a control group; i.e., the farming behavior of these herders is unlikely to be significantly influenced by the policy, making it theoretically viable to construct a control group. The rationale for using the contracted pasture area as the basis for grouping is as follows: The contracted pasture area for herders was determined exogenously by local governments in the 1980s. Consequently, this grouping variable is not associated with endogenous factors, such as grazing ability or risk tolerance, that may impact herders' farming efficiency. Therefore, utilizing the contracted pasture area as a grouping variable theoretically preserves the unbiasedness of the estimation results derived from the propensity score matching model.

Based on the aforementioned analysis, this paper employs Stata 17.0 to estimate propensity score matching for the baseline regression model. Table 6 presents the results of pastoral production efficiency and the average treatment effect on the treated (ATT) between the experimental and control groups, obtained through nearest neighbor matching (k=4), radius matching (0.01), and kernel matching. The results indicate that the estimates derived from these three matching methods are largely consistent, suggesting that the findings are robust. The average value of ATT is approximately 0.0702, which implies that the implementation of the GECP increases the pastoral production efficiency of herders by 0.0702 after controlling for confounding variables. These findings indicate that the GECP positively influences the breeding efficiency of herdsmen. The common support domain serves as a fundamental test objective for assessing the efficacy of the Propensity Score Matching (PSM) method. To ensure the validity of the matching results, it is essential to examine the common support domain of both the treatment and control groups. By analyzing the trend of the propensity score values for samples in both groups after applying the three matching methods, we can identify the overlap, which constitutes the common support domain (Fig 3). The findings reveal that most samples in both groups fall within the same range, with only a minimal number of samples being excluded after propensity score matching. This outcome increases the robustness of the common support domain in this study.

**Table 6. The Results of PSM.**

| **Matching Methods** | **ATT** | **S.E.** |
| --- | --- | --- |
| Nearest Neighbor Matching (K=4) | 0.0799** | 0.0349 |
| Radius Matching (0.01) | 0.0693** | 0.0344 |
| Kernel Matching | 0.0614* | 0.0331 |
| Mean Value | 0.0702 | - |

*** refers to the significance level of 1%.

**Fig 3. Kernel Probability Distribution after Propensity Score Matching.**

Finally, after matching, a balancing test was used to determine whether the variables were balanced between the control and treatment groups. The results of the balancing test are shown in Table 7. The Pseudo R2 value decreased from 0.094 before matching to 0.004~0.012. The LR statistic decreased from 39.07 to 0.90~2.90. The probability value changed from significant to insignificant. The mean deviation decreased from 19.5% to 3.30~6.20%. The median deviation decreased from 20.70% to 2.8~5.60%. In summary, the total error of the matched samples was greatly reduced, and the characteristics of the samples were similar between groups. The balance test was passed.

**Table 7. The Results of the Balance Test after Variable Matching.**

| Matching Methods | Pseudo R^2^ | LR Statistic | p Value | Mean Deviation | Median Deviation |
| --- | --- | --- | --- | --- | --- |
| Before Matching | 0.094 | 39.07 | 0.000 | 19.5 | 20.7 |
| Nearest Neighbor Matching (K=4) | 0.012 | 2.62 | 0.998 | 6.2 | 4.8 |
| Radius Matching (0.01) | 0.014 | 2.90 | 0.996 | 6.6 | 5.6 |
| Kernel Matching | 0.004 | 0.90 | 1.000 | 3.3 | 2.8 |

***[Comments 6]：In the DEA model's input-output indicators, the paper uses "herding income" (a flow indicator) and "year-end livestock stock" (a stock indicator) simultaneously as output variables. The scientific validity of this approach is questionable, and the authors should provide a sufficient scholarly justification for this choice.***

**[Response 6]：**Thank you for your rigorous and constructive suggestions. In this paper, we utilize a combination of two types of variables: herd income and year-end livestock stock. This approach is adopted to mitigate the potential bias associated with relying on a single indicator, thereby enhancing the accuracy of our research results. Specifically, herd income, which serves as the "economic flow" from current farming activities, encompasses transfer income from the sale of livestock, wool, and cashmere, as well as grassland ecological subsidies. This variable directly reflects the profitability of production activities during the current period. In contrast, year-end livestock stock represents the "stock of assets" related to farming activities, illustrating the unrealized reserves of production resources and the future output potential of herders.

Measuring the production capacity of herding households solely by "herd income" can lead to results that are easily influenced by external factors, such as fluctuations in market prices. Conversely, relying exclusively on "year-end livestock stock" fails to account for hidden factors, including market conditions, the quality of livestock breeds, and product premiums. The integration of both types of indicators provides a more comprehensive understanding and assists in identifying anomalies, thereby enhancing data quality assessment. For instance, if a herder reports a high inventory yet generates very low income from livestock farming, this discrepancy may indicate data misreporting (e.g., an inflated inventory figure) or special circumstances (e.g., inability to sell livestock due to epidemics). Conversely, if a herder has a low inventory but unusually high income, it is essential to investigate whether this is attributable to exceptional factors, such as the sale of rare breeds or substantial subsidies received.

This method has also been widely employed in existing studies. For instance, Qian (2019) utilized both "livestock stock at the end of the year" and "livestock income of herdsmen" as output indicators when analyzing the moderate scale of operation of herdsmen across different grassland types in Inner Mongolia. Similarly, Wang (2023) employed both "number of livestock slaughtered" and "livestock income" as indicators to examine the impact of operational scale on comprehensive efficiency and operational effectiveness.

In summary, the concurrent use of "livestock income" and "year-end stocking" as output indicators can significantly mitigate the risk of distortion associated with a single indicator, thereby enhancing the reliability and explanatory power of the calculation results.We also explain the selection of these two indicators in the article. Detailed changes can be found in lines 326-368 of the article

Reference

[1] Qian G X, Zhang N, Qian F M. Study on moderate scale of grassland animal husbandry under different target orientations: Based on herder data from four grassland types in Inner Mongolia[J]. Agricultural Economics and Management, 2019, (02): 55-66.
[2] Wang H, Du F L. Empirical study on the impact of herders' operation scale on comprehensive efficiency and economic benefits: A case study of typical grassland areas[J]. Journal of Agricultural Technical Economics, 2023, (08): 100-112. doi:10.13246/j.cnki.jae.2023.08.008.

Response to Reviewer 2 Comments

**Title：Impact of the grassland ecological compensation policy on pastoral production efficiency—evidence from pastoral China**

**Manuscript ID:** PONE-D-25-40838

Thank you for your meticulous revisions of our articles in your busy schedule, which have helped us immensely in improving the quality of our articles. We have revised the manuscript in accordance with the comments and marked all the amends on our revised manuscript. The reviewer comments are laid out below in *italicized font* and specific concerns have been numbered. Our responses are listed in red font, the specific modified contents are listed in blue font, and the modified contents are highlighted in yellow in the manuscript.

***[Comments 1]：Abbreviations must be defined at first mention (such as DEA, VRS, etc.).***

**[Response 1]:** Many thanks for your valuable suggestions, which have been extremely helpful in improving the overall quality of our manuscript. In response,We provided a detailed definition when the abbreviation was first mentioned.The full name of DEA is "Data Envelopment Anlysis".The full name of CRS is "Constant Returns to Scale". The full name of VRS is "Variable Returns to Scale".The specific modifications are as follows:

In lines 265-266, we change “The DEA model, also known as Data Envelopment Anlysis (DEA), is a nonparametric measure for evaluating the relative effectiveness of decision-making units. ” to “The Data Envelopment Analysis (DEA) model is a nonparametric measure for evaluating the relative effectiveness of decision-making units. ”

In lines 271-274, we change “The models include the CRS model with constant returns to scale and the VRS model with variable returns to scale, each of which can be subdivided into two categories: input-oriented and output-oriented. ” to “The models include the Constant Returns to Scale (CRS) model and the Variable Returns to Scale (VRS) model, each of which can be subdivided into two categories: input-oriented and output-oriented [31]. ”

***[Comments 2]：The description of the sample selection process is not detailed enough, and the representativeness of the sampling method needs further explanation.***

**[Response 2]:** We greatly appreciate the insightful changes you have suggested. In response, We have provided a more detailed description of the sample selection process and a more specific explanation of the representativeness of the sampling method. Specific changes can be made to lines 231-260 in the revised draft.The specific modifications are as follows:

The data used in this study come from field surveys conducted by the research group from January to May 2024 in the cities of Hulunbeier and Chifeng, Xilingol League and Ordos in Inner Mongolia, China. The survey area was selected with the following considerations. First, the Inner Mongolia Autonomous Region is located in northern China and has a long and narrow shape, which is usually divided into three major parts, namely, the eastern, central and western parts, based on its physical geography, climate and economic characteristics. Therefore, to ensure that the sample reflects the actual situation of Inner Mongolia as much as possible, the research team chose Hulunbeier and Chifeng in the eastern region, Xilingol League in the central region, and Ordos in the western region, for a total of four leagues (cities) as the research area. Second, the research area of pastoral industry development is relatively aggregated and specialized. Because the main target of the GECP is herders, the selection of the region where pastoral production is the main industry as the research object can accurately reflect the impact of the GECP on the production efficiency of herders.

This study adopts a combination of stratified sampling, typical sampling and random sampling methods. First, stratified sampling methods are used to select 9 pastoralist banners (counties), such as New Balkhuru Rouge Banner, Arukolqin Banner and Wengniuot Banner, from the league (city) according to the annual per capita net income of the county. Second, typical sampling methods are used to select 1~3 soums (towns) and 2~3 gacha (villages) per soum (township). Finally, 12~17 farmers (villages) are selected in each gacha (village) by simple random sampling. In this study, 510 questionnaires for herders and 47 questionnaires for villages (gacha) were distributed in the grass–animal balance area, and 468 valid questionnaires for herders were obtained after the questionnaires with seriously missing data were excluded, with an effective rate of 91.76%; 45 questionnaires for villages were valid, with an effective rate of 95.74%. The valid sample data cover 9 herding flags (counties), 19 soums (townships) and 45 villages (gacha).

***[Comments 3]：The chart titles and descriptions can be more detailed, and some tables lack necessary annotations. For instance, table 4, what do TE, PTE and SE stand for?***

**[Response 3]:** Thank you for your patience in reviewing this paper. In response to the comments, We have provided a more detailed explanation of the icon title and description. The specific modifications are as follows:

**Table 4. Efficiency Measurement Results.**

|  | **Conventional** | **Corrected** | **Bais** | **Lower limit** | **Higher limit** |
| --- | --- | --- | --- | --- | --- |
| TE^a^ | 0.48 | 0.43 | -0.06 | 0.41 | 0.45 |
| PTE^b^ | 0.53 | 0.49 | -0.13 | 0.42 | 0.52 |
| SE^b^ | 0.92 | 0.86 | -0.12 | 0.81 | 0.94 |

^a^TE represents technical efficiency.

^b^PTE represents pure technical efficiency.

^c^SE represents scale efficiency.

***[Comments 4]：The use of terminology needs to be more consistent (such as mixing 'herding households' and 'herders').***

**[Response 4]:** Thank you for your valuable comments. We changed "herding households" in the original text to "herders".

***[Comments 5]：The policy recommendation section can be more specific and more closely related to research findings.***

**[Response 5]：**We sincerely thank you for your insightful review and valuable comments on our findings. We have made more specific modifications to the policy recommendations section to make it more closely related to the research findings.The specific modifications are as follows:

Recommendations

(1)	Promote advanced breeding techniques to increase the efficiency of pastoral production. The government should enhance the socialized service system for animal husbandry and strengthen the relevance and accessibility of technology promotion to improve the generally low breeding efficiency observed among pastoral households. Concurrently, it is essential to actively conduct pastoral technology training to increase the overall quality of herdsmen, thereby aligning their skills with the demands of modern animal husbandry and increasing pastoral production efficiency.

(2)	Continuously improve the grassland ecological reward policy and its supporting policies. It is imperative to continuously refine the supporting policies of the GECP and facilitate the transformation and improvement of grassland animal husbandry. Governments at all levels must bolster the construction of basic security capabilities and increase policy support for essential components such as shed construction, high-quality forage base development, and the procurement of breeding machinery. These efforts should be systematically integrated into the framework of the ecological subsidy award policy to alleviate the capital constraints and technological bottlenecks that herders encounter during the initial stages of breeding method transformation. By increasing the controllability of herders in shelter-feeding breeding, we can effectively improve their breeding conditions, thereby promoting the successful transformation and improvement of grassland animal husbandry.

(3)	Guide herders to adopt appropriate scale management strategies. The government should establish differentiated standards for moderate-scale operations that align with the needs of herdsmen and the ecological conditions of grasslands. Furthermore, it is imperative to promote the concept of sustainable animal husbandry among herdsmen. Encouraging the formation of professional cooperatives, family ranches, and other organizational structures will facilitate the integration and optimization of production factors, including pasture, machinery, and labor. The aim of this approach is to increase resource allocation efficiency and ultimately improve breeding technology and enhance economic benefits.

***[Comments 6]：English writing requires further polishing, some sentences have grammatical errors and issues with expression fluency.***

**[Response 6]：**Thank you for your insightful comments on the policy recommendations section. We have made more professional polishing of the language of the article to reduce grammatical errors existing in the paper and improve its fluency of expression. The specific modification contents can be found in the revision traces in the revised draft.
